# Supplementary material for: Content-rich biological network constructed by mining PubMed abstracts
Source: BMC Bioinformatics. 2004 Oct 8;5:147. doi: 10.1186/1471-2105-5-147 (PMC528731; doi:10.1186/1471-2105-5-147)
Supplement: Additional File 2 — The original results of the above study (non-essential files are deleted to keep the file size under the limit set by BMC bioinformatics). [file 1471-2105-5-147-S2.bz2 › chilibotAdditionalFile2/dip10/97ID7768895E415/html/SNAP25_SYN3.html]

 


 **SNAP25** and **SYN3** 
  
Found 8 abstracts in PubMed, retrieved 8.  
 

 What does Google say? 
 PDF only 
| .edu only 

---

**Interactive relationship** (e.g. stimulation, inhibition, etc)

**Neutral relationship**- By immunoprecipitating syntaxin 1 depleted retina and brain extracts with a SNAP 25  [ **SNAP25** ]  antibody and microsequencing the precipitated proteins, syntaxin 3  [ **SYN3** ]  was detected in retina complexed with SNAP 25  [ **SNAP25** ] , synaptobrevin, and complexin.  Ref: 8824312 J Neurosci, 1996
- Analysis of protein extracts from brain regions shows that SNAP 25  [ **SNAP25** ] , syntaxin 1, and 4 are broadly distributed, while SNAP 23, syntaxin 3  [ **SYN3** ] , and 7 show distinct patterns of expression.  Ref: 10411979 Brain Res, 1999

**Non-interactive relationship** (e.g. studied together, co-existance, homology, etc.)

- ... syntaxin 3  [ **SYN3** ]  and SNAP 25  [ **SNAP25** ]  were phosphorylated by Ca2 and calmodulin dependent protein kinase II and cyclic AMP dependent protein kinase, respectively.  Ref: 9930733 J Neurochem, 1999
